# Description and life cycle of a new species of the genus *Arachnanthus* (Cnidaria: Anthozoa: Ceriantharia) from the Southwestern Atlantic Ocean

**DOI:** 10.7717/peerj.15290

**Published:** 2023-05-22

**Authors:** Celine S.S. Lopes, Fabrizio Scarabino, Alvar Carranza, Ricardo González Muñoz, André C. Morandini, Renato Mitsuo Nagata, Sérgio Nascimento Stampar

**Affiliations:** 1 Instituto de Biociências, Departamento de Zoologia, Universidade Estadual Paulista, Botucatu, São Paulo, Brazil; 2 Departamento de Ciências Biológicas, Faculdade de Ciências, Universidade Estadual Paulista, Bauru, São Paulo, Brazil; 3 Centro Universitario Regional del Este (Universidad de la República), Maldonado/Rocha, Uruguay; 4 Museo Nacional de Historia Natural, Montevideo, Uruguay; 5 Instituto de Investigaciones Marinas y Costeras (IIMyC), CONICET; Facultad de Ciencias Exactas y Naturales, Universidad Nacional de Mar del Plata, Mar del Plata, Argentina; 6 Departamento de Zoologia, Instituto de Biociências, Universidade de São Paulo, São Paulo, Brazil; 7 Centro de Biologia Marinha, Universidade de São Paulo, São Sebastião, São Paulo, Brazil; 8 Instituto de Oceanografia, Universidade Federal do Rio Grande, Rio Grande, Rio Grande do Sul, Brazil

**Keywords:** Tube-dwelling anemones, Taxonomy, Evolution, Life cycle, Temperate South America

## Abstract

**Background:**

Ceriantharia is a subclass of the phylum Cnidaria, which comprises tube-dwelling marine invertebrates. This subclass is composed of three families, including Arachnactidae, with two known genera. Currently, the genus *Arachnanthus* has five valid species recorded from Australia, the Mediterranean Sea and both the Southern and Northern Pacific Ocean. However, at the moment, there is no record of organisms of this family from the South Atlantic Ocean. Besides that, the life cycle of any species of the genus *Arachnanthus* is known. The present study describes a new species of the genus *Arachnanthus* and its life cycle, based on specimens from Uruguay and South of Brazil.

**Methods:**

Larvae were collected by plankton net in Rio Grande—Brazil and the development and external morphology of these specimens were observed in the laboratory during two years, and subsequently described. Additionally, nine adult ceriantharians correspondent to the larvae from Rio Grande were collected in Uruguay and their external and internal anatomies, and cnidome were described.

**Results:**

*Arachnanthus errans* **sp. nov.** exhibited a free-swimming, short-lived cerinula larvae that spent short-time on the plankton. The larva developed into small and translucent polyps with a short actinopharynx, one pair of mesenteries attached to a siphonoglyph, and a medium first pair of metamesenteries. Further, the adult polyp displayed an unprecedented locomotion behavior in Ceriantharia that is first reported here, it can crawl under and in between the sediment.

## Introduction

Ceriantharia is a small subclass of the class Anthozoa, commonly known as tube-dwelling anemones, which includes about 54 valid species (Stampar et al., 2020). Ceriantharians are morphologically distinguishable from other anthozoans by synthetizing a type of cnida, the ptychocyst, a unique feature of the group (Mariscal, 1974). Tube-dwelling anemones exhibit two ranks of external and internal tentacles, the marginal and labial tentacles respectively (Carlgren, 1912), and pores may occur throughout this structure (ØVan Beneden, 1924; Lopes et al., 2019). The organization of mesenteric filaments is also different in Ceriantharia, the trilobed shape of the filaments is uncertain; and new mesenteries emerge from the multiplication chamber (Stampar et al., 2016).

Moreover, ceriantharians have some distinct behavioral mechanisms from the other anthozoans; such as the building of a soft, mucous tube around the column (Child, 1908; Tiffon, 1987; Stampar et al., 2015a) important for their protection. So far, it is unknown if after the tube construction for any reason ceriantharians leave their tubes and crawl over the substrata. Tube-dwelling anemones can develop the following two patterns: (i) one more similar to the other Anthozoa, with the presence of a planula larva (Nyholm, 1943; Uchida, 1979) and, (ii) another, without the planula larva, but with the presence of a free-swimming larva, the cerinula, which can remain in the water column for up to 120 days (Stampar et al., 2015b), which is a unique arrangement among anthozoans. However, this general mechanism of the subclass is based only on some individuals, mainly of the family Cerianthidae Milne-Edwards & Haime, 1851.

Since the proposal of Den Hartog (1977), Ceriantharia is defined in two groups, Spirularia and Penicillaria, based on the composition of the cnidome (presence or absence of penicilli), arrangement of mesenteries (quartets or duplets), size of the hyposulcus, and presence or absence of acontioids and craspedonemes. Three families are known in Ceriantharia, Cerianthidae and Botrucnidiferidae (Carlgren, 1912) that compose Spirularia, and Arachnactidae (McMurrich, 1910) belonging to Penicillaria (Stampar et al., 2020), although it seems to be an underestimated number, as many taxonomic issues are observed between families of this subclass, which need an extensive revision (see Stampar et al., 2020). The Arachnactidae family is distinguished from the others by the arrangement of mesenteries in duplets (metamesenteries and betamesenteries), long hyposulcus and presence of acontioids (Carlgren, 1912; Den Hartog, 1977); and it is composed of two genera: *Arachnanthus* (Carlgren, 1912) and *Isarachnanthus* (Carlgren, 1924), the latter widely studied (Molodtsova, 2003; Stampar et al., 2012; Stampar et al., 2015b), unlike the former. The taxonomic history of Ceriantharia is full of inconsistencies, mainly in the family Arachnactidae. One of the incongruences was the description of species based solely on the morphology of larval forms even with little knowledge about the life cycle of ceriantharians and their patterns, which is currently still reflected in taxonomic studies and biodiversity estimates (Sars, 1846; Molodtsova, 2004).

The effectiveness of the approaches that consider only morphological characters of the ceriantharians in the larval stage is questionable, because they do not address the enormous phenotypic variation of the adult specimens already recognized for the group (Haime, 1854; ØVan Beneden, 1924; Nyholm, 1943; Uchida, 1979; Stampar et al., 2015b), such as the distinct mesenteric arrangement, the length of the protomesenteries, and the presence of directive tentacle, among others (Carlgren, 1912; Lopes et al., 2019). Furthermore, this approach does not use development data under an evolutionary perspective (*e.g.*, Stampar et al., 2015b).

The knowledge about the genus *Arachnanthus* is quite scarce; the clade has five valid species described that are represented by short organisms (2.5 to 4.2 cm) with translucent body and nocturnal habits (Carlgren, 1912; Carlgren, 1937). Besides that, these specimens use the tube as hiding places and quickly contract the entire body into it in response to any risk (CSS Lopes and SN Stampar, 2020, pers. obs.). Consequently, sampling of these ceriantharians is very difficult (Den Hartog, 1977; Stampar et al., 2018), which reflects the limited knowledge about systematics, developmental stages, evolutionary and ecological processes of the genus *Arachnanthus* (see Picton & Manuel, 1985; Carlgren, 1912; Molodtsova, 2004). The genus exhibits a wide distribution, ranging from the South of the Red Sea (Stampar et al., 2018), Australia (Carlgren, 1937), North Sea (Carlgren, 1912) and South and North Pacific Ocean (OBIS, 2022). Until the present day, there is no record of the genus *Arachnanthus* to the Southwestern Atlantic Ocean and there is no study published about life cycle, behavior and tube construction of this genus. Based on this scenario, we present the description of a new species of the genus *Arachnanthus* from the Southwestern Atlantic Ocean (Brazil and Uruguay), a description of its life cycle, based on wild collected planktonic larvae, and we record a new behavior of locomotion to a species of Ceriantharia.

## Material & Methods

The electronic version of this article in Portable Document Format (PDF) will represent a published work according to the International Commission on Zoological Nomenclature (ICZN), and hence the new names contained in the electronic version are effectively published under that Code from the electronic edition alone. This published work and the nomenclatural acts it contains have been registered in ZooBank, the online registration system for the ICZN. The ZooBank LSIDs (Life Science Identifiers) can be resolved and the associated information viewed through any standard web browser by appending the LSID to the prefix http://zoobank.org/. The LSID for this publication is: [urn:lsid:zoobank.org:pub:A2A48659-9BB7-4314-8F5A-1B0073A10246]. The online version of this work is archived and available from the following digital repositories: PeerJ, PubMed Central SCIE and CLOCKSS.

### Sampling of specimens

Hundreds of larvae were collected with the aid of a plankton net (mouth opening of 60 cm and mesh size of 300 µm) during winter (June to September) and summer (December to March) seasons of 2017 and 2018. Collection was conducted on the surf zone of the Cassino Beach, close to the mouth of the Patos Lagoon estuary, in Rio Grande, Rio Grande do Sul State, extreme south of Brazil (between 32°16′ to 32°22′S, and 52°18′ to 52°19′W). Nine adult specimens were collected during two expeditions of the Aldebaran and Miguel Oliver ships between 1995 and 2010, using a Mega Box Corer in Uruguay throughout the summer (December to March), on the boundary of the continental shelf at 124 and 248 m depth (between 35°56′24″S and 53°2′20″W; 36°9′46″S and 53°18′43″W). Eight of these specimens were preserved in 95% ethanol, and another was stored in formalin. All preserved adult ceriantharians from this study are deposited at the Zoology Museum of the USP—Brazil (MZUSP) and National Natural History Museum, Montevideo—Uruguay (MNHNM). Field collections were approved by Instituto Chico Mendes de Conservação da Biodiversidade—ICMBio and Sistema de Autorização e Informação em Biodiversidade—SISBIO (project number: 72673-1).

### Larval and adult rearing

Five larvae were maintained in a small aquarium (15 cm length X 10 cm width) with surface aeration and water circulation system. The water salinity and temperature were maintained between 30 to 34 and 22 to 26 °C, respectively, following ambient values we had measured in the field. The bottom was covered with a thin (∼3 cm) layer of sediment composed of sand and mollusk shells. The larvae were fed daily with newly hatched nauplii of *Artemia* sp. The development, including larval growth and settlement and adult growth, were filmed using a camera Sony HandyCam DCR-SR and described. After metamorphosis, three polyps were kept alive during two years and the behavior and morphological development were observed and described. Other larvae and adults were preserved to further analyses. 

### Morphological analysis

The external morphology of the larvae along with the development was described (following Stampar et al., 2015b). The morphological and cnidome studies of nine adult specimens were based on criteria adopted by Carlgren (1912) and Stampar et al. (2018). All adult ceriantharians were longitudinally dissected along the ventral side using surgical scalpels and a thorough study of each part of the animal’s body was performed under stereomicroscope. Protomesenteries/directive mesenteries, metamesenteries and betamesenteries were measured, and the proportion occupied by these mesenteries in the gastrovascular cavity was calculated (see: Lopes et al., 2019). The internal anatomy of the adult specimens described in this study was compared with the available literature (Cerfontaine, 1891; Carlgren, 1912; Carlgren, 1924; Carlgren, 1937; Stampar et al., 2018). Pseudocycles of the marginal and labial tentacles were verified considering the position of the tentacles in relation to the siphonoglyph (Carlgren, 1912). The number between parentheses means absence (0) or presence (1) of the directive labial tentacles. Subsequent numbers means the position of the tentacles.

The cnidome analyses were performed from measurements (length and width) of 30 undischarged capsules of each type of cnida from each body part (tip of the labial and marginal tentacles, actinopharynx, column, betamesenteries and metamesenteries) of the holotype and two paratypes (MZUSP 8727 and MNHNM 4299). Cnidae were visualized using Nikon Eclipse E200 microscope at 40X magnification and the photographs and measurements were carried out with Motic Images Plus 2.0 software. We followed the classification and nomenclature of the cnidae proposed by Mariscal (1974) and Stampar et al. (2018) with slight modifications. The distinction between types of cnidae was performed according to (i) size, shape and morphology of undischarged capsules; (ii) shape of shaft; and (iii) proportion occupied by shaft in the capsule.

During the collection, one adult specimen was separated and anesthetized using a solution of 5% MgCl2 with sea water. Posteriorly, this ceriantharian was preserved in formalin 10% with sea water (Spano & Flores, 2013). The specimen was dehydrated, incorporated in paraffin and histological sections of 6–10 µm were performed and stained with hematoxylin-eosin (Estrada, Peralta Zamora & Rivas Manzano, 1982).

## Results

### Systematics

**Table:** 

Phylum Cnidaria Verrill (1865)
Class Anthozoa Ehrenberg (1834)
Subclass Ceriantharia Perrier (1893)
Suborder Penicillaria Den Hartog (1977)
Family Arachnactidae Carlgren (1912)
Genus *Arachnanthus*Carlgren (1912)

**Diagnosis.** Arachnactidae with sterile protomesenteries, mesenteries grouped in duplets (M and B), long metamesenteries (M) fertile (presence of gametogenic tissue) and with a double mesenteric filament, short betamesenteries (B) sterile, with single, convoluted mesenteric filament; very long stomodeum; lacking a directive labial tentacle; cnidome with *p*-mastigophores and *b*-mastigophores (after Carlgren, 1912; Carlgren, 1924; Carlgren, 1937; Den Hartog, 1977).

**Type species.** *Cerianthus oligopodus* (Cerfontaine, 1891) (see: Stampar et al., 2020).

**Table:** 

**Valid species**
*Arachnanthus oligopodus* Cerfontaine (1891)
*Arachnanthus sarsi* Carlgren (1912)
*Arachnanthus bockii* Carlgren (1924)
*Arachnanthus australiae* Carlgren (1937)
*Arachnanthus lilith* Stampar et al. (2018)

***Arachnanthus errans* sp. nov.**

**Distribution of *Arachnanthus errans* sp. nov.** Southwestern Atlantic Ocean (Brazil − Rio Grande do Sul − and Uruguay)

***Arachnanthus errans* sp. nov.**

**Figure 1:**
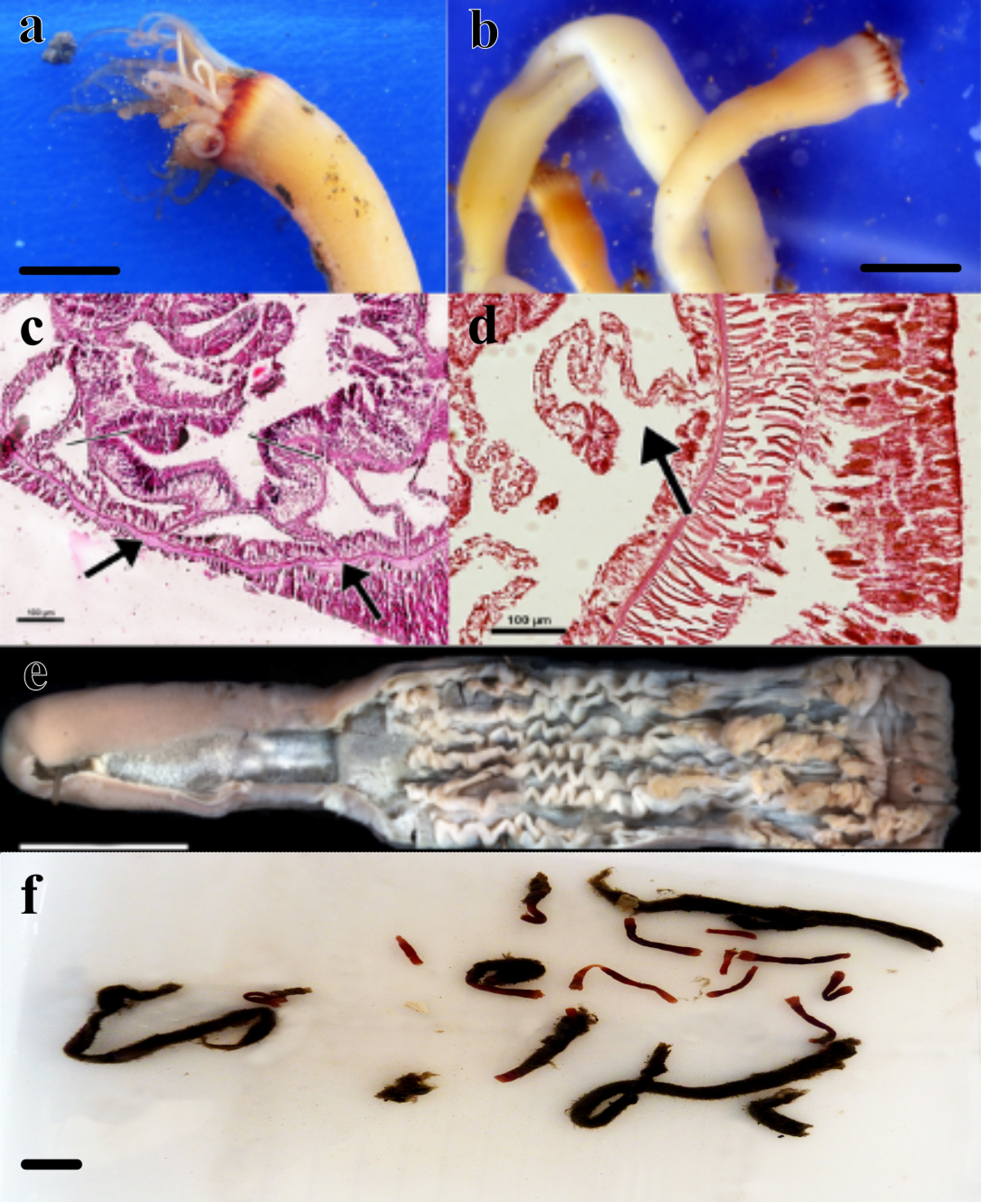
Specimens analyzed in this study. (A) color pattern of the live ceriantharian’s holotype tentacles after collection. Scale bar: 0.5 cm; (B) live ceriantharians after collection. Scale bar: 0.5 cm; (C) histological section of the pharynx region, the arrows indicate the siphonoglyph and pair of mesenteries connected to it; (D) directive mesentery attached to a siphonoglyph; (E) holotype ventraly dissected. Scale bar: 0.8 cm; (F) preserved paratypes and other ceriantharians of the same species stored in ethanol 90% to analysis. Scale bar: 0.3 cm.

**Figure 2:**
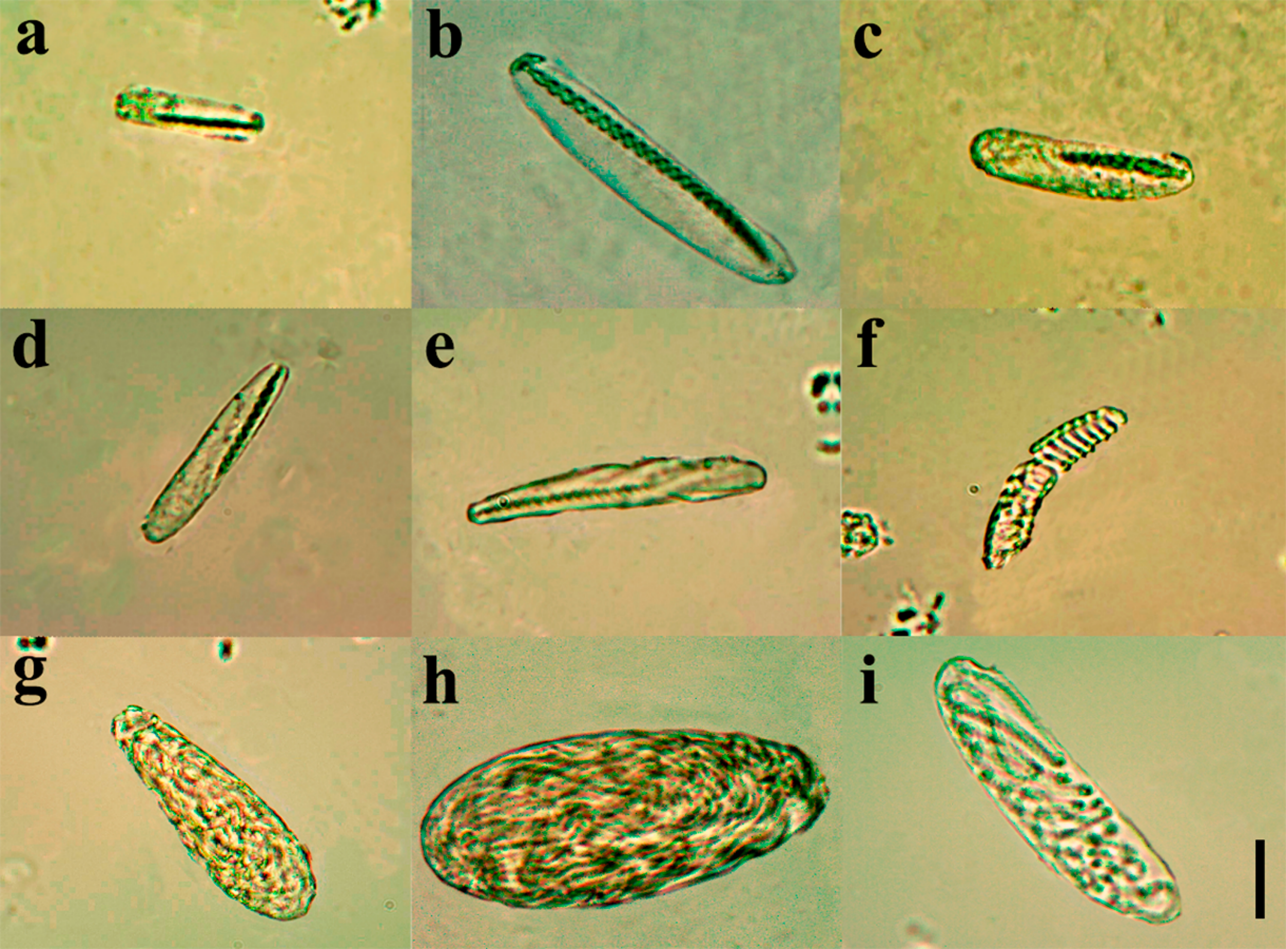
Cnidome at 100X magnification of three adult specimens of *A. errans* sp. nov.: holotype, paratypes MZUSP 8727 and MNHNM 4299. (A) early stage of microbasic *b*-mastigophore type I; (B) late stage of microbasic *b*-mastigophore type I; (C) early stage of microbasic *b*-mastigophore type II; (D) late stage of microbasic *b*- mastigophore type II; (E) microbasic *b*-mastigophore type III; (F) spirocyst; (G) Atrichous; (H) Ptychocyst, (I) Holotrichous. Scale bar: 10 µm.

**Figure 3:**
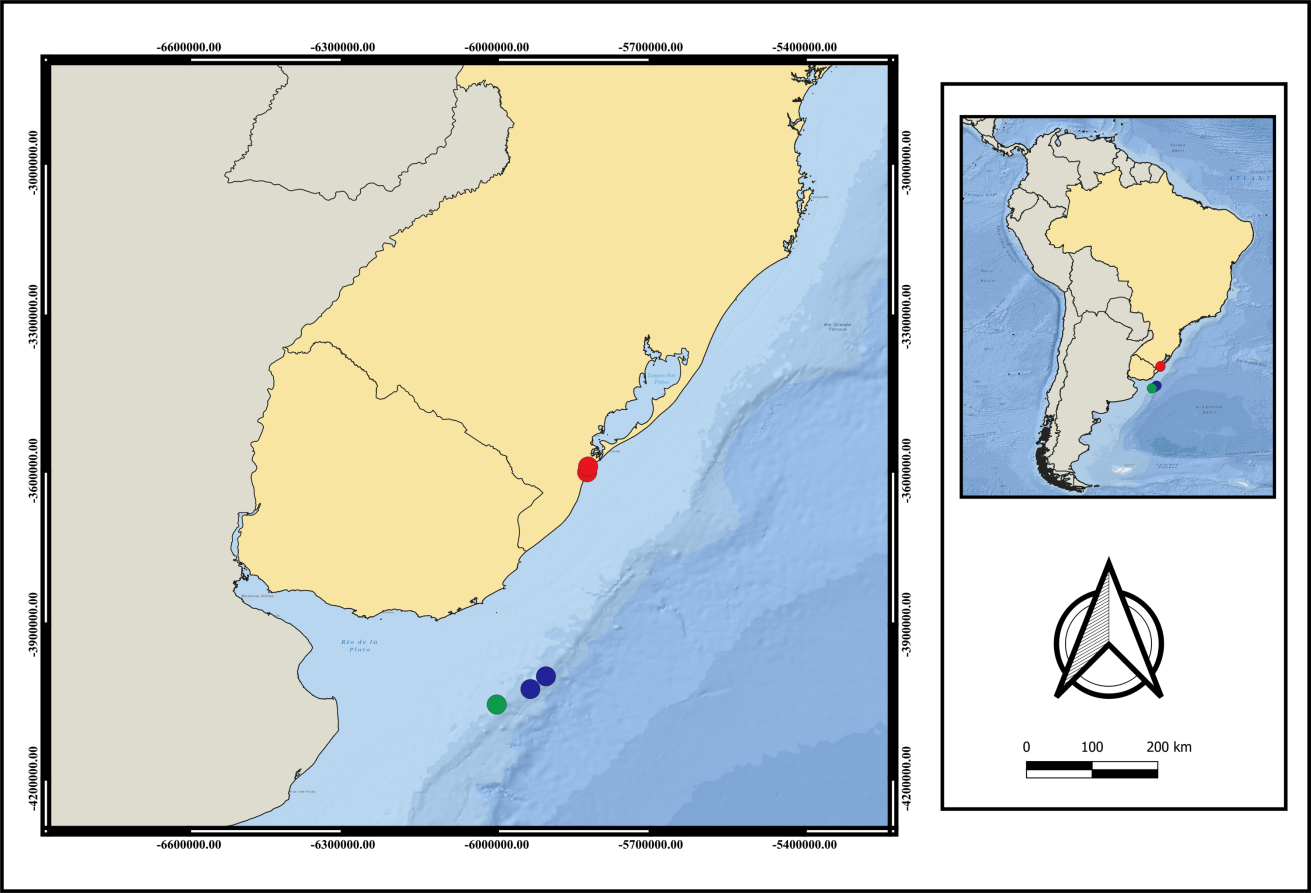
Map of distribution of *A. errans* sp. nov. Blue circles: Uruguay; red circles: Rio Grande—RS—Brazil and green circle: locality of holotype’s collection. Map created in QGIS 3.16.0 software. Download of the layers in shapefile format in: The Humanitarian Data Exchange.

**Figure 4:**
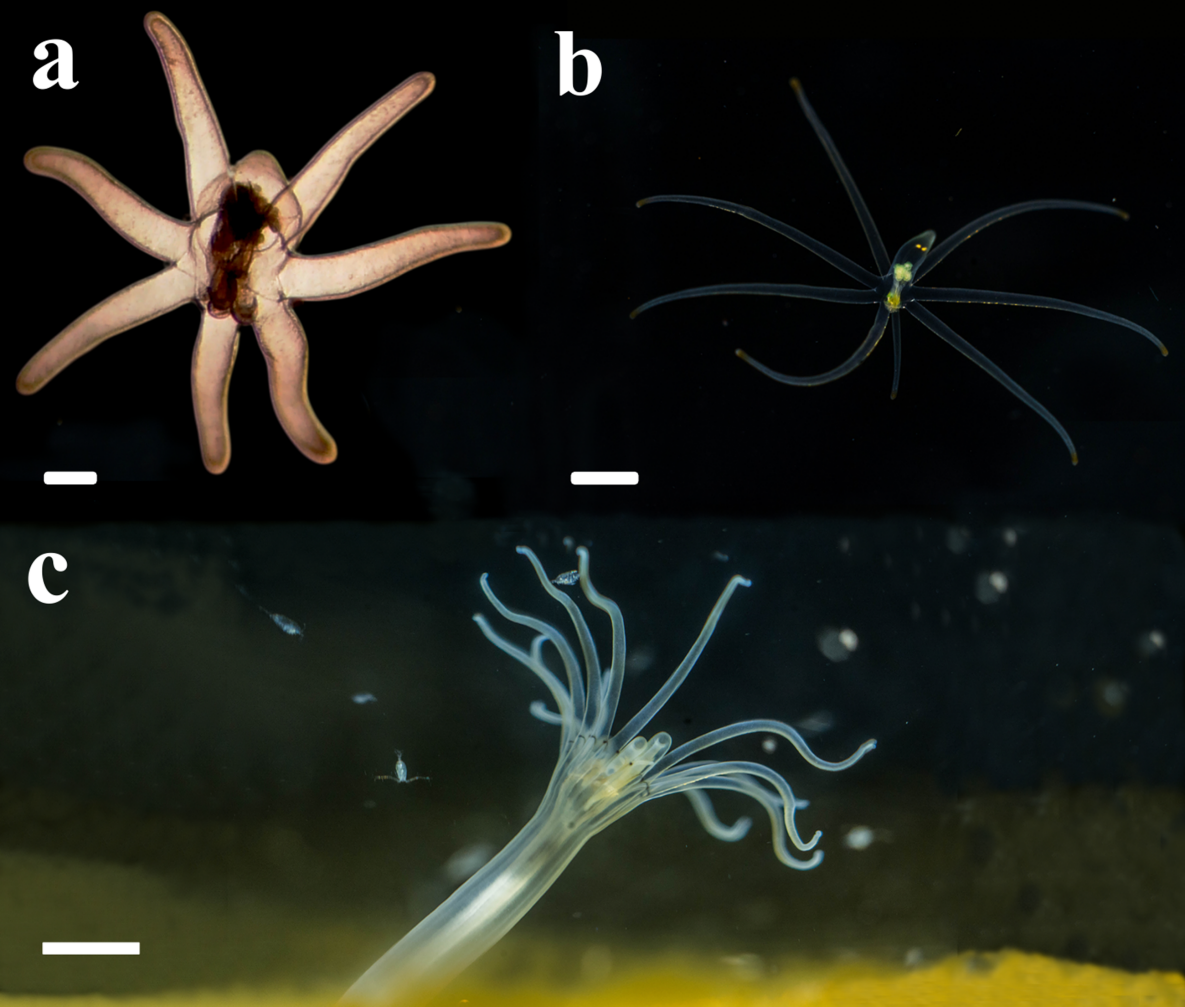
Life cycle of *A. errans* sp. nov. from Rio Grande—Brazil. (A) early live larva with ovoid body. Scale bar: 0.1 cm; (B) late live larva close to settlement. Scale bar: 0.3 cm; (C) live adult specimen after build the tube. Scale bar: 0.5 cm. Figure credit: Guilherme Morsch von Montfort.

**Figure 5:**
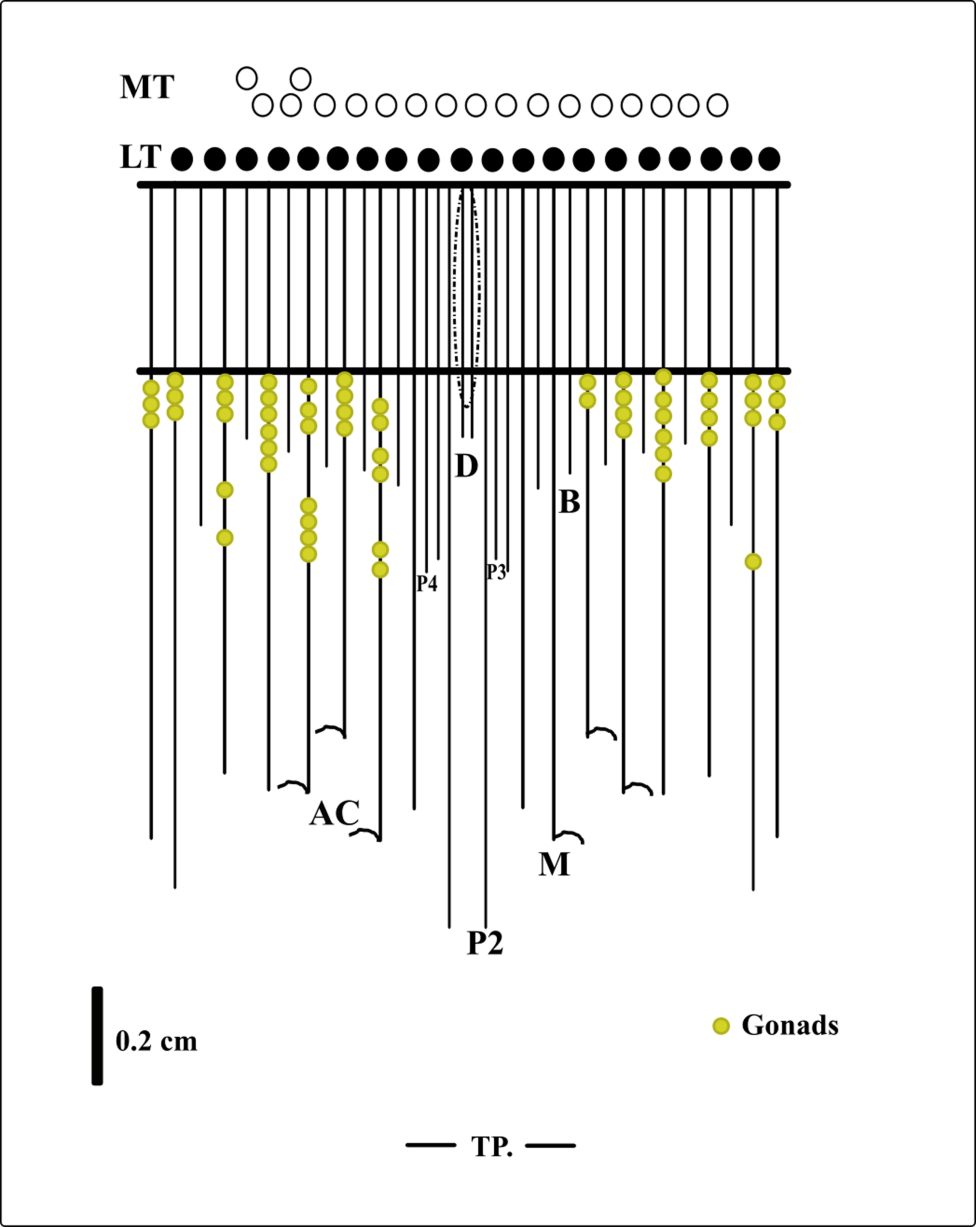
Graphical representation of the arrangement of mesenteries in the holotype of *A. errans* sp. nov. Abbreviations: MT, Marginal tentacles; white circles represent the insertion of each marginal tentacle. LT, Labial tentacles; black circles represent the insertion of each labial tentacle. D, directive mesenteries. P2, second pair of protomesenteries. P3, third pair of protomesenteries. P4, fourth pair of protomesenteries. M, metameseteries. B, betamesenteries. AC, Acontioids. TP, terminal pore.

**Figure 6:**
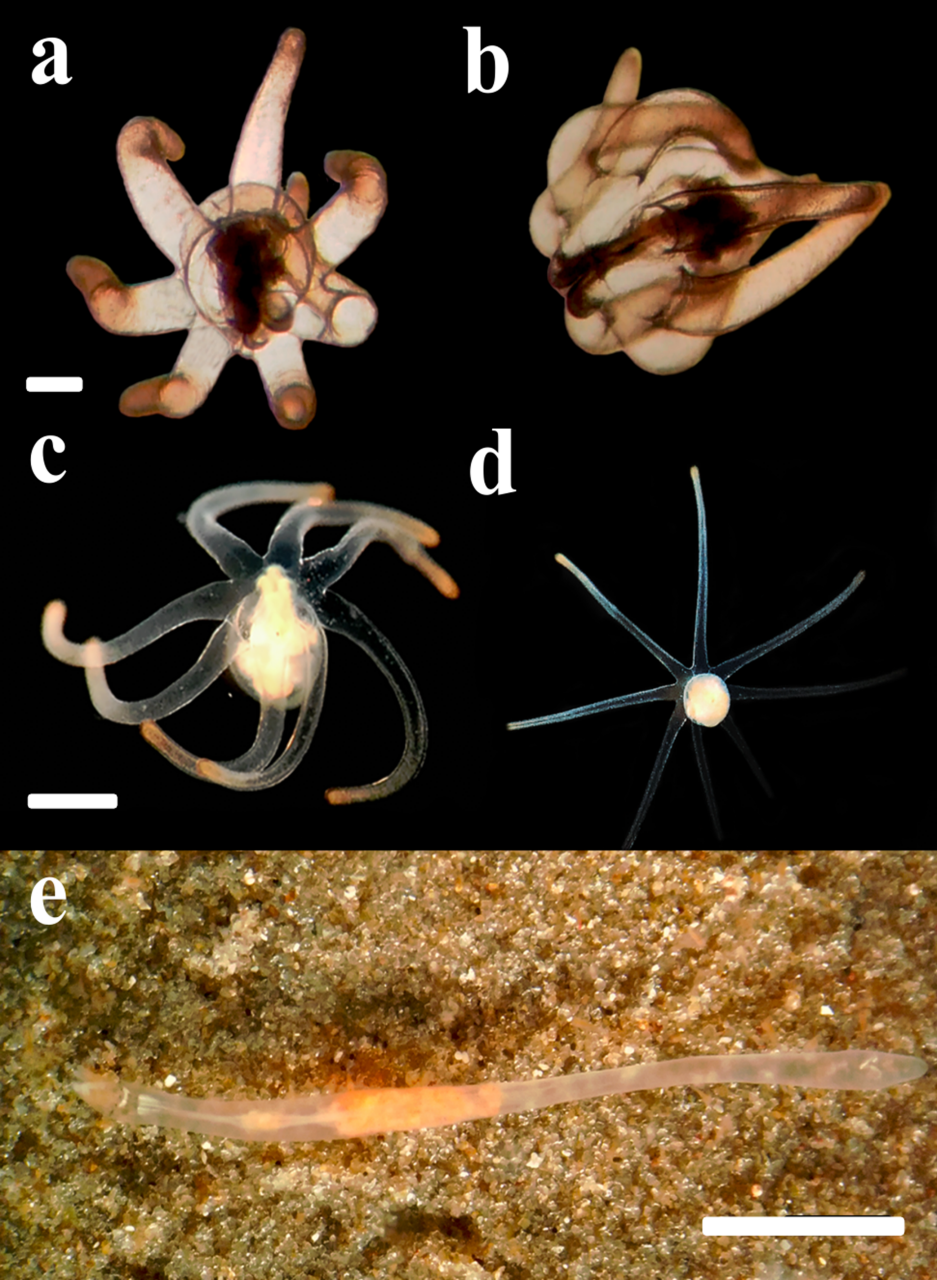
Behavior of the larvae and adult specimens from Rio Grande—Brazil. (A) Free-swimming early larva; (B) marginal tentacles toward to column. Scale bar (A) and (B) 0.1 cm; (C) rotational movement of the late larva with mouth facing to up; (D) late larva floating with mouth facing down. Scale bar (C) and (D) 0.3 cm; (E) specimens displaying “errantia life” behavior and crawling on the sediment. Scale bar: 0.5 cm.

Zoobank link: urn:lsid:zoobank.org:act:BD7C4EEF-670F-4B18-9BC1-70FA1C9895B8

(Figs. 1–6, Tables 1–3)

**Material examined (nine specimens, Fig. 1). Holotype:** MNHNM 4296 **•** adult individual, 2.84 cm long and 0.58−0.77 cm wide, Uruguay (124 m depth − 36°26′S, 53°54′W − salinity and temperature 33.7 and 7.2 respectively), M. Barona collector (07/x/1995) (R/V Aldebaran Cruise 9508 st.34). **Paratypes:** MZUSP 8727 **•** adult individual, 1.92 cm long and 0.1−0.2 cm wide, Uruguay (239 m depth − 36°9′46″S, 53°18′43″W), Alvar Carranza collector (04/ii/2010); MZUSP 8728 **•** damaged adult individual (tissue of body slightly torn vertically), ∼1.54 cm long and 0.38 cm wide, Uruguay (248 m depth − 35°56′24″S, 53°2′20″W), Alvar Carranza collector (01/ii/2010); MZUSP 8729 **•** adult individual, 1.85 cm long and 0.50 cm wide, Uruguay (239 m depth − 36°9′46″S, 53°18′43″W), Alvar Carranza collector (04/ii/2010); MZUSP 8730 **•** damaged adult individual (tissue of body slightly torn vertically), 2.01 cm long and 0.39 cm wide (the width was measured at the height of actinopharynx), Uruguay (248 m depth − 35°56′24″S, 53°2′20″W), Alvar Carranza collector (01/ii/2010); MZUSP 8731 **•** damaged adult individual (harmed marginal tentacles; some of them was squashed and lost during collection), 1.38 cm long and 0.33−0.40 cm wide, Uruguay (239 m depth − 36°9′46″S, 53°18′43″W), Alvar Carranza collector (04/ii/2010); MNHNM 4297 **•** damaged adult individual (tissue of body torn vertically), 3.07 cm long and 0.38 cm wide, Uruguay (239 m depth − 36°9′ 46″S, 53°18′43″W), Alvar Carranza collector (04/ii/2010); MNHNM 4298 **•** damaged adult individual (tissue of body torn vertically), ∼1.89 cm long, Uruguay (239 m depth − 36°9′ 46″S, 53°18′43″W), Alvar Carranza collector (04/ii/2010); MNHNM 4299 **•** adult individual, 5.2 cm long and 0.32−0.59 cm wide, Uruguay (248 m depth − 35°56′24″S, 53°2′20″W), Alvar Carranza collector (01/ii/2010).

**Diagnosis.** Small ceriantharian with short actinopharynx and hyposulcus. One pair of mesenteries connected to a siphonoglyph, short directives mesenteries—DM (Figs. 1C–1D), second pair of protomesenteries (P2) long range half of gastrovascular cavity length. First pair of metamesenteries (M1) smaller than gastrovascular cavity length. Acontioids on second, third and fourth pair of metamesenteries (M2, M3 and M4), Cnidome (Fig. 2) of spirocysts, microbasic *b*-mastigophore (two types), holotrichous, atrichous and ptychocysts (Table 1).

**Distribution.** Currently only recorded from Southwestern Atlantic- Brazil, Rio Grande do Sul State and Uruguay (Fig. 3). The adults were only found between 124 m to 248 m depth. Larvae were collected only during the day.

**Etymology.** The specific name “*errans*” is related to the remarkable locomotion behavior in the adult phase, in which specimens frequently leave the tube and move under and in between substrates.

**Live color.** Translucent body and light brown column along body length, transparent marginal tentacles with brown tips, brownish base between marginal tentacles. Labial tentacles whitish with the basal part brown, surrounding the white oral disc (Figs. 1A–1B and 4C).

**Description of holotype.** (MNHNM 4296—Figs. 1A and 1E). Small hermaphrodite ceriantharian with 2.84 cm long and 0.77 (width measured at the height of the actinopharynx) −0.58 cm wide (width measured at the height of the initial part of the gastrovascular cavity = GC). Approximately 15 marginal tentacles arranged in one pseudocycle (0)1.21.21.11.11…, pores following a horizontal line along tentacles length. 11 labial tentacles with 0.13 cm long arranged in one pseudocycle (1)1.11.11.11…, directive tentacle present. Small actinopharynx, 0.17 cm long and 0.77 cm wide, occupies 5.9% of total body length, siphonoglyph elongated, 0.21 cm long and 0.13 cm wide, one pair of mesenteries attached to a siphonoglyph. Hyposulcus short (0.05 cm long) and hemisulci present. Gastrovascular cavity large with 2.45 cm long and 0.58 (at the initial part) − 0.70 cm (at the final part) in diameter, occupies 86.2% of the total body length. Mesenteries arranged in couples of metamesenteries (M) and betamesenteries (B) (Fig. 5). Four pairs of protomesenteries, including directive mesenteries (DM). DM very short with 0.08 cm, P2 long with 1.87 cm and P3 and P4 short with 0.60 and 0.64 cm respectively. DM, P2, P3 and P4 occupy 3.2, 76.3, 24.4 and 26.1% respectively of the total GC length. Metamesenteries (M) fertile with double mesenteric filament, three first pairs of metamesenteries occupy 45, 46.1 and 40.1% respectively of the GC length. Betamesenteries (B) sterile and short with double mesenteric filament only in initial part, three first pairs of betamesenteries occupy 13.3, 12.3 and 11.6% respectively of the GC length. Acontioids present. Cnidome of spirocysts, microbasic *b*-mastigophore (two types), holotrichous, atrichous and ptychocysts (Table 1).

**Comparison between specimens of *A. errans*** **sp. nov.** Small ceriantharians with translucent body, 1.38−5.2 cm long, 0.1−0.77 cm wide. 13–16 translucent marginal tentacles (0.14−0.32 cm long) arranged in (0)1.21.21.11.11…with almost all brown tips and pores along each tentacle. 6–10 translucent labial tentacles (0.07−0.13 cm long) with the brown base (Figs. 1A–1B), arranged in (1)1.11.11.11…or (0)1.11.11.11…; pores present, directive tentacle present or absent. Short actinopharynx (0.10−0.41 cm long) occupies 5.2−7.8% of the total body length; short hyposulcus (0.05−0.10 cm long) and hemisulci distinct; siphonoglyph larger than actinopharynx length (0.21−0.34 cm long and 0.08−0.14cm wide), one pair of mesenteries connected to siphonoglyph. Large GC (1.25−4.9 cm long, 0.14−0.7 cm wide) occupies 81.1%-94.2% of the total body length, short directive mesenteries (0.08−0.19 cm long), second pair of protomesenteries (P2) long with 0.54−4.0 cm long, P3 and P4 short with 0.6 and 0.64 cm long, respectively, metamesenteries (M) medium, fertile and with double mesenteric filament, occupy more than 46% of GC, short and sterile betamesenteries (B), occupy less than 28% of GC, acontioids present.

**Comparison with other members of the genus.** *Arachnanthus errans* sp. nov. has labial tentacles arranged in one pseudocycle like *A. sarsi*, *A. oligopodus* and *A. australiae* and distinct from *A. lilith* which has labial tentacles arranged in three pseudocycles. The amount of pseudocycles of the labial tentacles in *A. bockii* is uncertain and can be arranged in one or two. The actinopharynx of *A. errans* sp. nov. occupies from 6 to 7% of the gastrovascular cavity length, whereas in *A. bockii*, *A. oligopodus* and *A. sarsi* occupies 50%, *A. australiae* 66% and in *A. lilith* it occupies less than the gastrovascular cavity length. *A. errans* sp. nov. has one pair of mesenteries connected to a siphonoglyph different to other members of the genus, *A. sarsi* has three pairs, *A. lilith* with four pairs of mesenteries, *A. australiae* and *A. bockii* both with six pairs of mesenteries, and *A. oligopodus* has two pairs of mesenteries attached to a siphonoglyph. M1 extent almost to the aboral pore in *A. australiae*, *A. bockii* and *A. sarsi*, in *A. oligopodus* and *A. lilith* reach the aboral pore and in *A. errans* sp. nov. can reach about 52% of the GC length. The acontioids were in the M2, M3 and M4 unlike all others (Table 2).

**Table 1:** Measurements of 30 capsules for each cnida type in all body regions of the holotype and two paratypes (MZUSP 8727 and MNHNM 4299) of *A. errans* sp. nov. (*N* = 3). Numbers inside parentheses indicate cnidae length and width, respectively, and numbers outside parentheses indicate average cnidae size. N/N, number of individuals for which each type of cnida was found/number of specimens for which cnidae were measured.

**Body part/cnida type**	***Arachnanthus errans***	**N/N**
**Marginal tentacles**		
Microbasic *b*-mastigophore type I (early stage)	28.0 (21.7-34.3) × 5.5 (3.8–7.2)	3/3
**Labial tentacles**		
Microbasic *b*-mastigophore type I (early stage)	24.05 (15.3-32.8) × 4.05 (2.7–5.4)	2/3
Microbasic *b*-mastigophore type I (late stage)	40.1 (35.2-45.0) × 6.4 (5.4–7.4)	3/3
Microbasic *b*-mastigophore type II (late stage)	35.35 (31.8-38.9) × 6.0 (4.9–7.1)	3/3
**Pharynx**		
Microbasic *b*-mastigophore type I (early stage)	25.1 (20.8-29.4) × 5.0 (3.7–6.3)	3/3
Microbasic *b*-mastigophore type II (early stage)	22.5 (18.2-26.8) × 3.4 (2.5–4.3)	3/3
Microbasic *b*-mastigophore type II (late stage)	36.35 (30.9-41.8) × 6.1 (5.4–6.8)	3/3
**Column**		
Atrichous	43.85 (30.0-57.7) × 16.8 (10.6–23.0)	3/3
Microbasic *b*-mastigophore type I (early stage)	29.5 (26.0-33.0) × 7.4 (5.7–9.1)	1/3
Microbasic *b*-mastigophore type II (early stage)	29.85 (23.2-36.5) × 6.8 (5.8–7.8)	3/3
Microbasic *b*-mastigophore type VII	37.5 (33.0-42.0) × 4.55 (3.3–5.8)	1/3
Ptychocyst	71.2 (62.0-80.4) × 26.95 (22.2–31.7)	3/3
Holotrichous	54.8 (50.0-59.6) × 5.75 (5.0–6.5)	2/3
**Mesenteries M**		
Microbasic *b*-mastigophore type I (early stage)	21.9 (16.2-27.6) × 4.55 (2.5–6.6)	3/3
Microbasic *b*-mastigophore type I (late stage)	45.65 (35.8-55.5) × 8.65 (6.0–11.3)	2/3

**Table 2:** Compilation of the main morphological characters of the genus *Arachnanthus* (after Carlgren, 1912; Carlgren, 1924; Carlgren, 1937; Picton & Manuel, 1985; Stampar et al., 2018).

	*A. errans sp. nov.*	*A. lilith*	*A. oligopodus*	*A. sarsi*	*A. australiae*	*A. bockii*
Marginal tentacles	Up to 16	Up to 14	∼20	Up to 35	Up to 40	Up to 30
Arrangement of the labial tentacles	(1)1.11.11.11 or (0)1.11.11.11	(0)3.12.31.23.23.12	(0)1.11.11.11.11	(0)1.11.11.11.11	(0)1.11.11.11.11	(0)1.11.11.11.11(?)
Lenght of the actinopharynx	6.2-6.9% of the GC	>50% of the GC	∼50% of the GC	∼50% of the GC	66% of the GC	∼50% of the GC
Hyposulcus	24–29% of the actinopharynx	Same length of the actinopharynx	2X the length of the actinopharynx	Smaller than length of the actinopharynx	∼50% of the actnopharynx	∼50% of the actnopharynx
Number of mesenteries connected to siphonoglyph	2	8	4	6	12	12
Directive mesenteries	3.2–3.8% of the GC (<length of the actinopharynx)	<length of the actinopharynx	>length of the actinopharynx	<length of the actinopharynx	= length of the actinopharynx	<length of the actinopharynx
Proportion of the P2 length in the GC	76% of the length GC	85% of the length GC	50% of the length GC	75% of the length GC	50% of the length GC	25% of the length GC
Proportion of the P3 length in the GC	24–35% of the size GC	33% of the size GC	∼50% of the size CG	∼33% of the size GC	<25% of the size GC	<25% of the the size GC
Proportion of the M1 length in the GC	52% of the length GC	To aboral pore	To aboral pore	Almost to aboral pore	Almost to aboral pore	Almost to aboral pore
Proportion of the M3 length in the GC	53–79% of the length GC	75% of the length GC	50% of the length GC	Almost to aboral pore	80% of the length GC	Almost to aboral pore
Cnidoglandular tract of B	Present	Present	Present	Present	Present (short?)	Present (short?)
Cnidoglandular tract of the fertile mesenteries	Present (short)	Present (short)	Present (short?)	Present (short)	Present (short?)	Present (short?)
Acontioids	M2, M3 and M4	M3 and M4	M1	M1, M2, M3	M1, M2, M3	M1, M2, M3

**Notes.**

GC gastrovascular cavity DM mesenteries directives P2 second pair of protomesenteries P3 third pair of protomesenteries M1 first pair of metamesenteries M2 second pair of metamesenteries M3 third pair of metamesenteries

Literature: *A. sarsi* in Carlgren (1912); *A. bockii* in Carlgren (1924); *A. australiae* in Carlgren (1937); *A. oligopodus* in Picton & Manuel (1985); *A. lilith* in Stampar et al. (2018).

### Description of life cycle

Filming of the development stages was performed during ten days and additional records were made along two years, in which adults specimens were kept alive in the laboratory. A total of 523 min of the development course of *A. errans* sp. nov. were recorded. Some these materials were used to assemble a short video (Video S1).

### Larval stage

Larvae with ovoid-shaped body, with a swollen middle section, while the distal part was slightly sharp and elongated (Fig. 4A). The larval body was translucent and the length of preserved organisms varied between 0.4 cm and 0.7 cm. The length of tentacles is about the same as the column length in smaller individuals. However, a disproportionate lengthening of the column occurred through larval development. Early larvae exhibited seven well-developed and transparent marginal tentacles with the brownish tips. The central mouth aperture is surrounded by three, to usually four, small labial tentacles. Late larvae developed an eighth shorter tentacle and rudiments of a fifth labial tentacle (Fig. 4B). Protruding manubrium, marginal tentacles were perpendicular to the aboral region. The pharynx and directive mesenteries were visible due to the color of the body. The gastrovascular cavity was formed; mesenteric filaments were strongly marked and occupied about half or more than half of this region. After some days, the length of the body increased, ranging from 1.0 cm to 1.6 cm (preserved organism) and exhibited a more conical shape, with the distal region more elongated. Some late larvae collected from the field with some sand grains attached to the middle section of their column, however this habit was not observed in our cultures. The actinopharynx and gastrovascular cavity was more extended, protomesenteries were apparent and acontioids at the end of one pair of metamesenteries were visible in the terminal pore. As the larva matures, it darkens in color and becomes thicker. The mesenteric filaments were less pronounced concentrated only in the proximal part of the gastrovascular cavity.

### Larval behavior

The marginal tentacles were active most of the time in the early larvae, alternating between slower movements at the level of the oral disc and rapid movements towards the basal part. A relatively fast lowering of the marginal tentacles may propel the larvae through the water column (Figs. 6A–6B), but this behavior was occasional. In addition to this swimming behavior, the larvae can float and rotate on the water surface with their mouth downwards and facing perpendicularly. The late larval body sometimes floated in the water column with the mouth facing downwards, or eventually with the mouth turned up rotating the body and tentacles (Figs. 6C–6D). The epidermal ciliary beat that generates iridescence was visible at higher magnification, against a dark background. Finally, shortly before settling, the larvae open the oral region and catch the prey that was offered to them, the labial tentacles helped to bring the prey to the mouth, however, the larva regurgitated; it turned the body up, rotating and contracting the body until the *Artemia* sp. was externalized through the manubrium.

### Settlement

Before the settlement, larvae changed the posture of the marginal tentacles from laterally extended, with the tips curved downwards, to distally aligned, parallel to the body length and, slightly contracted. Wild late-developed larvae found with numerous sand grains attached to the column may represent a pre-settling behavior that was not observed in the laboratory. This followed an extension of the body that became twice as long as the tentacle length. Along with such morphological transition, larvae completely lost their buoyancy and were found crawling over the bottom. Although the tip of the column was usually an anterior region during this crawling locomotion, larvae were also able to go backwards and seemed to be exhibiting a searching behavior for an adequate settlement site. Larvae spent about four days to one week to settle on the sediment. It burrows the sediment and the polyp builds a thin tube around the column, using sand and ptychocysts. There were evident morphological changes from larva and polyp forms, the main occurring in the column, which became relatively thin and elongated. The body exhibited milky color; however the transparency was maintained in some regions, mainly at the gastrovascular cavity. Around the oral disc, the polyp had light brown spots and at the base, between marginal tentacles there were dark brown stripes.

### Crawling and feeding behavior

After burrowing the sediment and consolidating its tube, the polyp may exhibit a remarkable crawling behavior, named ‘*errantia life’*. The polyp left the tube and crawled over the surface of the sediment (Figs. 6E–6F), and other times it buried and wandered inside the sediment, building tunnels, similar to the burrowing behavior of earthworms (Table 3). Although in most observations with natural light the adults remain hidden in their tubes, after being offered *Artemia* sp. nauplii, they quickly came out and remained feeding for a few hours. Marginal tentacles were stretched like a whip to catch the prey. The ceriantharian sometimes can catch the prey with the aid of the tip of marginal tentacles, or eventually by the middle part of the tentacles in a fast bending of the tentacle toward the oral region; the labial tentacles aid in this process, pushing the prey into the mouth. After the transfer of the prey to the mouth, marginal tentacles were extended and remained immobile. We observed the adult of the *A. errans* sp. nov. feeding on nauplii of *Artemia* sp. and copepods.

**Table 3:** Description of the “errantia life” behavior of *A. errans* sp. nov.

**Number of times that behavior was exhibit**	**Behavior**	**Description of the behavior**
–	Semi-sessile polyp behavior.	The adult polyp was inside the tube (built on substrate) with the body erect, a part of the body and tentacles were outside of the tube. The tentacles were not open and the crown of tentacles moved sometimes.
2		
6	Start of the body lateral movement.	The ceriantharian started to move the aboral axis from side to side.
2	Start body withdrawal of the tube.	During the movement of the entire body from side to side, the polyp left the tube through the front part.
2	Start the ‘*errantia life*’ behavior.	The adult ceriantharian totally left the tube and started the behavior of drag through the sediment.
2	Active ‘*errantia life*’ behavior.	*A. errans* sp. n. throughout adult life exhibited a behavior of crawling under and between the substrate.
2	Polyp immersed to the tube.	The semi-sessile polyp instead of leaving the tube by the front part immersed the body to within the tube and left it, probably, through the posterior part.
1	Start crawled the body between sediment.	The polyp crawled the body, probably by body contraction, between sediments.
–	Build of the new tube.	The adult built the new and thin tube with ptychocysts, mollusk shell and seabed sediments.

## Discussion

Taxonomic studies are essential to understand ecosystems, the relationships established between and within taxa, the biogeography of organisms, and the development of species conservation strategies (Costello & Chaudhary, 2017; Thomson et al., 2018). However, studies with marine invertebrates, mainly those that inhabit deep waters, are difficult to carry out, due to the inhospitality of the environment and the lack of habitat assessment (Costello et al., 2010). This scenario is intensified for small marine organisms from the deep sea, and infaunal animals, such as *Arachnanthus errans* sp. nov. (124–248 m depth). Arai (1965) had already pointed out this obstacle in studying the subclass Ceriantharia. Nowadays, difficulties to collect and study small marine invertebrates, with nocturnal habits and inhabit deep sea, such as those the genus *Arachnanthus* is maintained and can be reflected in the number of species described, only six, in the census of marine biodiversity (Costello et al., 2010) and in the amount of research groups dedicated to study tube-dwelling anemones. Therefore, studies like this are relevant to better know the infaunal marine invertebrates from Temperate South America, to reveal the development mechanisms inherent to these marine invertebrates, since this is the first life cycle described for a species of the genus *Arachnanthus*, and to discuss about evolutionary aspects of the development of the Ceriantharia.

There are records of some genera of Ceriantharia from the Southwestern Atlantic Ocean such as *Ceriantheomorphe* Carlgren, 1931 (Spier, Stampar & Prantoni, 2012), *Isarachnanthus* (Carlgren, 1924) (Stampar et al., 2012; Stampar et al., 2015b), *Ceriantheopsis* (Carlgren, 1912; Stampar et al., 2015b) *Cerianthus* Delle Chiaje, 1841 and *Pachycerianthus* (Roule, 1904; Stampar, Morandini & Silveira, 2014). *Arachnanthus errans* sp. nov. is the second species until now recorded from Rio Grande do Sul (Brazil) and Uruguay, the another species being *Ceriantheomorphe brasiliensis* (Mello-Leitao, 1919) (Lopes et al., 2019; Stampar et al., 2020). However, *Ceriantheopsis lineata* Stampar et al., 2015a and Stampar et al., 2015b, recorded from Southeast Brazil and Buenos Aires Province (Argentina), may occur in the same region because the area considered here is within these localities (Stampar et al., 2015a; Stampar et al., 2015b). Furthermore, two additional undescribed species belonging to genera *Cerianthus* and *Pachycerianthus* are under description and were collected between other localities from the Uruguayan coast (Stampar et al., 2012).

**Figure 7:**
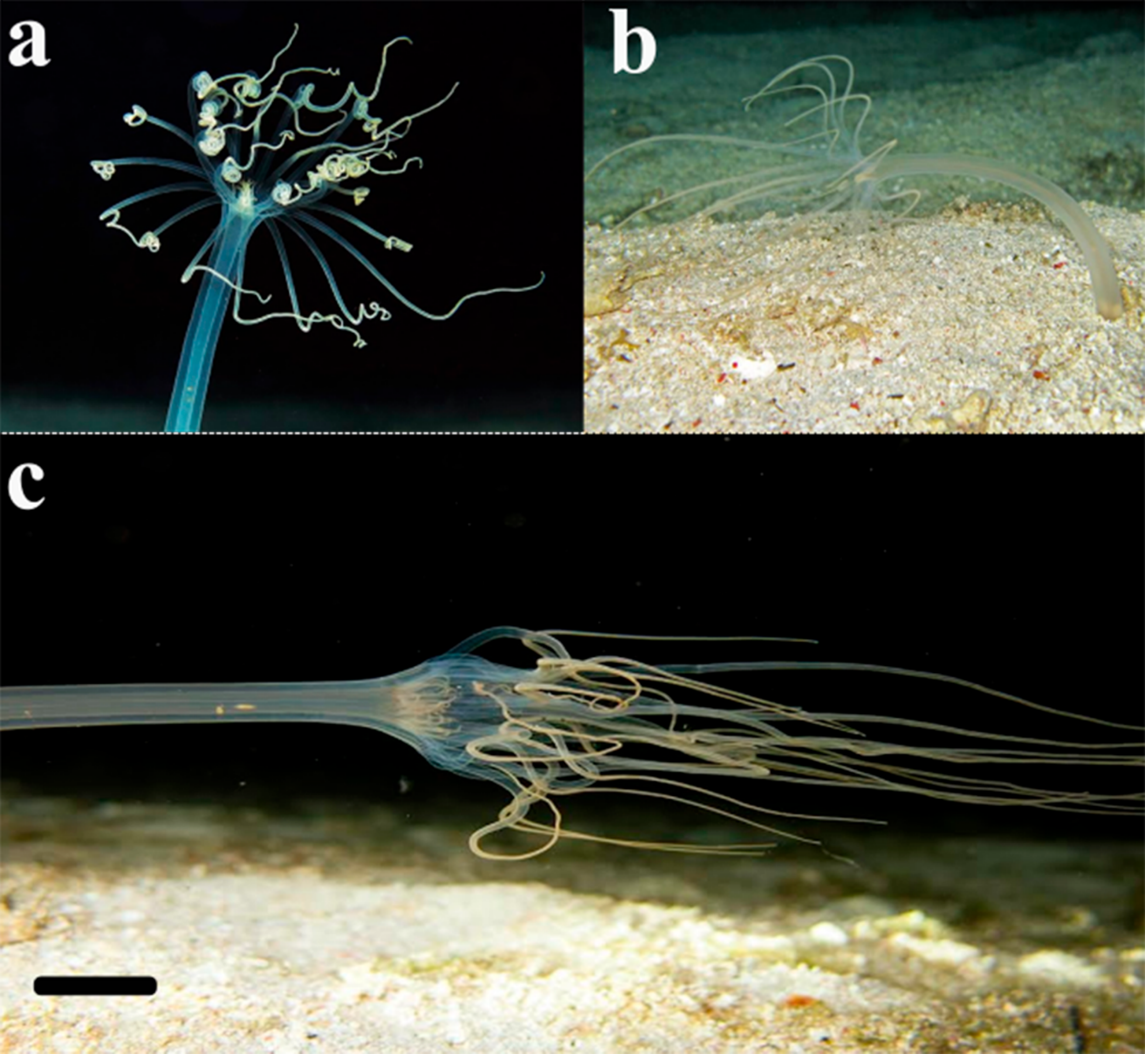
Record of the ceriantharian with external morphology similar to *A. errans* sp. nov. from the Caribbean region. Scale bar: 0.5 cm. Figure credit: Mickey Charteris.

The present study is the first record of the genus *Arachnanthus* from the Southwestern Atlantic Ocean. Larvae here described were found only in the summer season, when water temperature was between 23 and 25 °C and salinity between 31 and 34, all exhibited the same pattern of life cycle. Larvae that probably belongs to *A. errans* sp. nov. were previously found in 2% of plankton samples of a time series from the surf-zone, collected from 2009 to 2017 in the type location (Teixeira-Amaral et al., 2021). Those individuals were also found only during summer and within the same intervals of temperature and salinity. Moreover, there are photographic records of adult organisms that exhibit an external anatomy similar to *A. errans* sp. nov. from the Caribbean region (16°21′27″N, 86°55′60″W: Fig. 7), in a shipwreck area at 15m depth, in the silty sediment; but the specimens were not collected. However, if the record is confirmed, biogeographical and evolutionary discussions will be needed, since the distance between these regions is very large, more than 6.000 km, and the life cycle observed for *A. errans* sp. nov. is very short, similar to the pattern registered for other anthozoans (Fautin, 1991; Miller & Ball, 2000; Bocharova, 2016), but with a planktonic larva.

Cnidarians have different developmental routes, one of the reasons for the ecological success of the clade (Kayal et al., 2018), with groups exhibiting metagenesis, the Medusozoa, and other that have only the larval and polyp stage (Miller & Ball, 2000; SN Stampar, 2018, pers. obs.). Phylogenomic studies indicated that the evolutionary history of development stages in Cnidaria is extremely plastic, which makes it difficult the establishment of patterns (Piraino et al., 2004; Kayal et al., 2018; Leclère et al., 2019), some groups of cnidarians reveal alternative development routes (Stampar et al., 2015b; Matsumoto, Piraino & Miglietta, 2019). Throughout evolutionary history, ceriantharians developed two different mechanisms in the life cycle. The tube-dwelling anemones may have a short-time development with the presence of a planula larva similar to others anthozoans (Nyholm, 1943). Otherwise, instead of the planula, it may has a free-swimming larva named cerinula (Van Beneden, 1897; McMurrich, 1910; Molodtsova, 2003) that can remains in the water column for a relatively long time (Haime, 1854; Stampar et al., 2015b). This cerinula is able to disperse over longer distances and colonize different habitats (Lopes et al., 2023). Species such as *A. errans* sp. nov. has a simple cerinula larva with a shorter planktonic stage, which can be a limiting to range long distances, even if these larvae exhibit epidermal ciliary beat that may play an important role in the transport of surrounding fluid and in the locomotion. It was recently observed that ceriantharians of the same species can exhibit both long and short planktonic stages according to environmental changes related to temperature. The time of the larva in the plankton can originate adults with significant morphological variations, such as distinct arrangement of mesenteries (SN Stampar, 2019, personal observations). The plasticity in life cycles, and its macroevolutionary and microevolutionary implications are largely discussed in Medusozoa and explainable because of the metagenetic pattern of development (Boero & Sarà, 1987; Cunha, Maronna & Marques, 2016), including records of morphological variations that have conducted to taxonomic inconsistencies through taxa (Marques, 1995; Miglietta, Schuchert & Cunningham, 2009; Cunha, Maronna & Marques, 2016). However, anthozoans do not have metagenesis in the life cycle (Fautin, 1991) and understanding the plasticity in the morphological characters is challenging, which have corroborated with taxonomic incongruities and underestimated the diversity of species (Spier, Stampar & Prantoni, 2012; Stampar et al., 2012; Lopes et al., 2019). During nineteenth and twentieth centuries, the use of only larval morphology was quite recurrent to describe species in Ceriantharia, mainly those of the genus *Arachnanthus* (Sars, 1846; McMurrich, 1910). This practice generated a long list of unduly species described; most of them persisting until nowadays (see WoRMS, 2023, Stampar et al., 2020). Description of species only based on morphological traits of larvae was pointed out as inappropriate by Van Beneden (1897), but the practice was kept for a long time. Despite of the fact that ‘drastic’ changes do not occur in the morphology of the cerinula larva in comparison to adults of the ceriantharians, our results demonstrate increase and addition of structures in the adult stage, such as the number of tentacles and mesenteries, and elongation of the column. Therefore, a thorough taxonomic revision of the ceriantharian’s species should be performed (see Lopes et al., 2023).

The effectiveness of approach using morphology of the adult to identify Ceriantharia’s species is poorly discussed (Stampar et al., 2015b; Lopes et al., 2019). However, Fautin et al. (2007) defended that using only morphological traits of the adult to distinguish species of the genus *Arachnanthus* is inefficient, whereas Stampar et al. (2018) pointed out that since there were no records of the cryptic species for the genus, until then the internal anatomy of the adult was sufficient to differentiate species. Our analysis reveals the occurrence of intraspecific variation in three internal morphological characters of the species *A. errans* sp. nov., which actually are used as taxonomic markers: the absence of the directive tentacle, the length of P3 and M3 in relation to gastrovascular cavity. Furthermore, *A. errans* sp. nov. exhibit other characters distinct from its congeners, such as, small actinopharynx, the short hyposulcus, one pair of mesenteries connected to siphonoglyph, and M1 occupies more than half of gastrovascular cavity, but not extent at the aboral pore (Table 1). Our analysis agrees with Stampar et al. (2018), the junction of information about mesenteries can be appropriate to distinguish species in the genus *Arachnanthus* until the moment. Although *A. errans* sp. nov. exhibits variations in some mesenteries, others can be used to distinguish this species, such as, amount of mesenteries connected to a siphonoglyph, length of directive mesenteries, length of M1 and P2 and proportion that these mesenteries occupy in the gastrovascular cavity. Besides that, the short hyposulcus and actinopharynx can be used in addition to the mesenteries.

The tiny polyp of the *A. errans* sp. nov. (Fig. 4C) revealed a behavior never before registered for Ceriantharia: after larval settlement, metamorphosis to the polyp and tube formation, the ceriantharian completely left the tube and crawled under and between the sediment. The ability of dispersion is one of the strategies developed by organisms to respond to environmental cues (Brooker et al., 2007). For benthic marine species, the dispersion is mostly associated to the earliest development stage, such as the larval phase, to which is assigned the population connectivity (Cowen & Sponaugle, 2009). However, locomotory behavior exhibited by adult semi-sessiles, their evolutionary and ecological role are poorly understood, even for those taxa whose locomotion capacity is already known, such as sea anemones (McClendon, 1906; Parker, 1916). The adult sea anemone exhibits a crawling behavior on the substrate through waves of muscular contractions (Parker, 1916), which in *Exaiptasia diaphana* (Rap, 1829) is highly stimulated by periods of starvation (Bedgood et al., 2020). However, the behavior we found for the adult tube-dwelling anemone *A. errans* sp. nov. resembles the movement behavior of the some planula larvae in cnidarians, such as the hydroid *Clava multicornis* (Forsskål & Niebuhr, 1775), which crawls its entire body in worm-like shape on the substrate (Piraino et al., 2011; Pennati et al., 2013), except that *A. errans* sp. nov. can also burrow itself into the substrate and crawl among it. The behavior of burying the body and moving in the sediment was observed in *Echinoptilum* sp., a species of sea pen (Kushida, Higashiji & Reimer, 2020). Mushroom coral species completely flip-over the body onto the sediment (Hoeksema & Bongaerts, 2016). The mobile behavior of these corals is an ability developed for dispersal and reaching empty spaces, to get away from other corals (Chadwick, 1988) and toxic sponges (Hoeksema & De Voogd, 2012). The ecological reason for the peculiar behavior of *A. errans* sp. nov. is not yet clear and more studies need to be performed. However, exhibiting active movement behavior would be useful for protection and can be one of reasons to explain only one type of cnida—microbasic *b*-mastigophore - found in the marginal tentacles of the specimens analyzed in this study, since cnidocysts are important tools against predators. The behavior of withdrawal into the sediment that sea pens such as *Pennatula rubra* (Ellis, 1764) exhibit, is a response to environmental disturbances; in this process the anthozoan do not displace it in the seabed (Chimienti, Angeletti & Mastrototaro, 2018).

Ceriantharians are tube builder invertebrates; mostly, the tubes are described as flexible and soft structures that are built from combining filaments of ptychocysts and sediment or mud (Child, 1908; Stampar et al., 2015a). The tube is built in between the sediments (Tiffon, 1987) and play the role of sheltering the ceriantharian, protecting it from predators (Stampar et al., 2015a), but it can also serve as refuge to other small marine invertebrates, such as Amphipoda (Ceriello et al., 2021). The construction, length and shape of the tube by ceriantharians can be distinct and consists in relevant implications on the lifestyle. Organisms of the family Arachnactidae use the filaments of ptychocysts to adhere more sediment through the tube formation and have branched tubes, unlike the pattern observed to the Cerianthidae (Stampar et al., 2015a). Our results indicate that probably when displaying the ‘errantia life’ behavior, *A. errans* sp. nov. permanently leaves its tubes. Consequently, it can synthetize more than one tube through the lifespan, similar to other congeners of Arachnactidae, corroborating with observations of Den Hartog (1977) and, roam above and among sediment. Probably this behavior is related to building of a thinner and simpler tube by *A. errans* sp. nov., since specimens build other tubes along the life.

## Conclusions

Our analysis revealed that adult specimens of *A. errans* sp. nov. have one pair of directive mesenteries connected to a siphonoglyph, the stomodaeum length is small and the first pair of metamesenteries reached half of the gastrovascular cavity length. Besides that, the cnidome found in marginal tentacles was composed by only one type of cnida. The new species *Arachnanthus errans* sp. nov. was described from Southwestern Atlantic Ocean (South of Brazil and Uruguay); the first record of the genus *Arachnanthus* from this region. The life cycle of the species was observed and described. *Arachnanthus errans* sp. nov. has free-swimming larva of short-time (∼7 days). After settlement and construction of the mucous and thin tube, the adult definitively left the structure and crawled the entire body over the substrate, sometimes buried the entire body in the sediment and crawled under it; behavior that we called ‘*errantia life*’. The adult most likely builds more than one tube during its lifespan, because we did not observe the ceriantharian returning to the old tube. Locomotory behavior similar to the exhibited by *A. errans* sp. nov. adults are described to other cnidarians and are associated with a defensive process. The functional and ecological role of this crawling behavior should be better investigated, however, it is possible that along evolutionary history, *A. errans* sp. nov. developed another defense mechanism in addition to the synthesis of cnida and tube building habits.

##  Supplemental Information

10.7717/peerj.15290/supp-1 Video S1 Life cycle of the *Arachnanthus errans* sp.nov

## Additional Information and Declarations

### Field Study Permissions

The following information was supplied relating to field study approvals (*i.e.*, approving body and any reference numbers): Field experiments were approved by Instituto Chico Mendes de Conservação da Biodiversidade - ICMBio (project number: 72673-1).

### Data Availability

The following information was supplied regarding data availability: Specimens analyzed and described in this article are stored at the Zoology Museum of the USP - Brazil (MZUSP): MZUSP 8727, MZUSP 8728, MZUSP 8729, MZUSP 8730, MZUSP 8731 and at the and National Natural History Museum, Montevideo—Uruguay (MNHNM): MNHNM 4296, MNHNM 4297, MNHNM 4298, MNHNM 4299.

### New Species Registration

The following information was supplied regarding the registration of a newly described species: Arachnanthus errans sp. nov. urn:lsid:zoobank.org:act:BD7C4EEF-670F-4B18-9BC1-70FA1C9895B8 Publications LSID: urn:lsid:zoobank.org:pub:A2A48659-9BB7-4314-8F5A-1B0073A10246

## References

[ref-1] Arai MN (1965). A new species of *Pachycerianthus*, with a discussion of the genus and an appended glossary. Pacific Scientific.

[ref-2] Bedgood SA, Bracken MES, Ryan WH, Levell ST, Wulff J (2020). Nutritional drivers of adult locomotion and asexual reproduction in a symbiont-hosting sea anemone *Exaiptasia diaphana*. Marine Biology.

[ref-3] Bocharova E, Goffredo,S and Dubinsky Z (2016). Reproduction of sea anemones and other Hexacorals. The Cnidaria: past, present and future.

[ref-4] Boero F, Sarà M (1987). Motile sexual stages and evolution of Leptomedusae (Cnidaria). Bollettino di Zoologia.

[ref-5] Brooker RW, Travis JMJ, Clarck EJ, Dytham C (2007). Modelling species’ range shifts in a changing climate: the impacts of biotic interactions, dispersal distance and the rate of climate change. Journal of Theoretical Biology.

[ref-6] Carlgren O (1912). Ceriantharia. The Danish Ingolf-Expedition.

[ref-7] Carlgren O (1924). Papers from Dr. Th. Mortensen’s Pacific expedition 1914-16. XVI. Ceriantharia. Videnskabelige meddelelser fra Dansk Naturhistorisk Forening i København.

[ref-8] Carlgren O (1937). Ceriantharia and zoantharia. Scientific Reports of the Great Barrier Reef Expedition 1928.

[ref-9] Cerfontaine P (1891). Notes préliminaires sur l’organisation et le développement de différentes formes d’Anthozoaires. IV. Sur un nouveau cerianthe du golfe de Naples, Cerianthus oligopodus (n. sp.). Bulletin de la Classe des sciences.

[ref-10] Ceriello H, Senna AR, Andrade LF, Stampar SN (2021). Crustacea biodiversity in tubes of Ceriantharia (Cnidaria; Anthozoa), including the description of a novel species of Amphipoda from southeastern Brazil. Marine Biodiversity.

[ref-11] Chadwick NE (1988). Competition and locomotion in a free-living fungiid coral. Journal of Experimental Marine Biology and Ecology.

[ref-12] Child CM (1908). Form regulation in *Cerianthus aestuarii*. Biology Bulletin.

[ref-13] Chimienti G, Angeletti L, Mastrototaro F (2018). Withdrawal behaviour of the red sea pen *Pennatula rubra* (Cnidaria: Pennatulacea). The European Zoological Journal.

[ref-14] Costello MJ, Chaudhary C (2017). Marine biodiversity, biogeography, deep-sea gradients, and conservation. Current Biology.

[ref-15] Costello MJ, Coll M, Danovaro R, Halpin P, Ojaveer H, Miloslavich P (2010). A census of marine biodiversity knowledge, resources, and future challenges. PLOS ONE.

[ref-16] Cowen RK, Sponaugle S (2009). Larval dispersal and marine population connectivity. Annual Review of Marine Science.

[ref-17] Cunha AF, Maronna MM, Marques AC (2016). Variability on microevolutionary and macroevolutionary scales: a review on patterns of morphological variation in Cnidaria Medusozoa. Organisms Diversity & Evolution.

[ref-18] Delle Chiaje S (1841). Descrizione e notomia degli animali invertebrati della Sicilia Citeriore osservati vivi negli anni.

[ref-19] Den Hartog JC (1977). Descriptions of two new Ceriantharia from the Caribbean region. Pachycerianthus curacaoensis n. sp. and *Arachnanthus nocturnus* n. sp. with a discussion of the cnidom and of the classification of the Ceriantharia. Zoologische Mededeelingen.

[ref-20] Ehrenberg CG (1834). Beiträge zur physiologischen kenntnis der corallenthiereim allgemeinen, und besonders des rothen Meeres, nebsteinem versuche zur physiologischen systematik der selben. Abhandlungen der Königlich Preussischen Akademie der Wissenschaften.

[ref-21] Ellis J (1764). An account of the sea pen, or *Pennatula phosphorea* of Linnaeus; likewise a description of a new species of sea pen, found on the coast of South-Carolina, with observations on sea-pens in general. Philosophical Transactions of the Royal Society.

[ref-22] Estrada E, Peralta Zamora LE, Rivas Manzano P (1982). Manual de técnicas histológicas (No. 571.5.E8M3).

[ref-23] Fautin DG (1991). Developmental pathways of anthozoans. Hydrobiologia.

[ref-24] Fautin DG, Hickman CP, Daly M, Molodtsova T (2007). Shallow-water sea anemones (Cnidaria: Anthozoa: Actiniaria) and tube anemones (Cnidaria: Anthozoa: Ceriantharia) of the Galápagos Islands. Pacific Science.

[ref-25] Forsskål P, Niebuhr C (1775). Descriptiones animalium, avium, amphibiorum, piscium, insectorum, vermium/quae in itinere orientali observavit Petrus Forskål. Post mortem auctoris edidit Carsten Niebuhr. Adjuncta est materia medica kahirina atque tabula maris Rubri geographica.

[ref-26] Haime J (1854). Mémoire sur le cérianthe *Cerianthus membranaceus*. Annales des Sciences Naturelles.

[ref-27] Hoeksema BW, Bongaerts P (2016). Mobility and self-righting by a free-living mushroom coral through pulsed inflation. Marine Biodiversity.

[ref-28] Hoeksema BW, De Voogd NJ (2012). On the run: free-living mushroom corals avoiding interaction with sponges. Coral Reefs.

[ref-29] Kayal E, Bentlage B, Pankey MS, Ohdera AH, Medina M, Plachetzki DC, Collins AG, Ryan JF (2018). Phylogenomics provides a robust topology of the major cnidarian lineages and insights on the origins of key organismal traits. BMC Evolutionary Biology.

[ref-30] Kushida Y, Higashiji T, Reimer JD (2020). First observation of mole-like burrowing behavior observed in a sea pen. Marine Biodiversity.

[ref-31] Leclère Ł, Horin C, Chevalier S, Lapébie P, Dru P, Peron S, Jager M, Condamine T, Pottin K, Romano S, Steger J, Sinigaglia C, Barreau C, Artigas GQ, Ruggiero A, Fourrage C, Kraus JEM, Poulain J, Aury JM, Wincker P, Quéinnec E, Technau U, Manuel M, Momose T, Houliston E, Copley RR (2019). The genome of the jellyfish *Clytia hemisphaerica* and the evolution of the cnidarian life-cycle. Nature Ecology & Evolution.

[ref-32] Lopes CSS, Ceriello H, Morandini AC, Stampar SN (2019). Revision of the genus *Ceriantheomorphe* (Cnidaria, Anthozoa, Ceriantharia) with description of a new species from the Gulf of Mexico and northwestern Atlantic. Zookeys.

[ref-33] Lopes CSS, Maronna MM, Martinelli-Filho JE, Morandini AC, Stampar SN (2023). New evidence to demystify the supposed holoplanktonic life cycle in Ceriantharia (Cnidaria). Marine Biodiversity.

[ref-34] Mariscal RN, Muscatine L, Lenhoff HM (1974). Nematocysts. Coelenterate biology-reviews and new perspectives.

[ref-35] Marques AC (1995). Eudendrium pocaruquarum n. sp. (Hydrozoa, Eudendriidae) from the southeastern coast of Brazil, with remarks on taxonomic approaches to the family Eudendriidae. Contributions to Zoology.

[ref-36] Matsumoto Y, Piraino S, Miglietta MP (2019). Transcriptome characterization of reverse development in *Turritopsis dohrnii* (Hydrozoa, Cnidaria). G3 Genes, Genomes, Genetic.

[ref-37] McClendon JF (1906). On the Locomotion of a Sea Anemone (*Metridium marginatum*). The Biological Bulletin.

[ref-38] McMurrich JP (1910). Actiniaria of the Siboga expedition. Part I. Ceriantharia. Siboga-Exped.

[ref-39] Miglietta MP, Schuchert P, Cunningham CW (2009). Reconciling genealogical and morphological species in a worldwide study of the family Hydractiniidae (Cnidaria, Hydrozoa). Zoologica Scripta.

[ref-40] Miller DJ, Ball EE (2000). The coral Acropora: what it can contribute to our knowledge of metazoan evolution and the evolution of developmental processes. BioEssays.

[ref-41] Milne-Edwards H, Haime J (1851). Archives du Muséum d’Histoire Naturelle. Archives du Muséum d’Histoire Naturelle.

[ref-42] Molodtsova TN (2003). On *Isarachnanthus* from Central Atlantic and Caribbean region with notes on *Isarachnactis lobiancoi* (Carlgren, 1912). Zoologische Verhandelingen.

[ref-43] Molodtsova T (2004). On the taxonomy and presumable evolutionary pathways of planktonic larvae of Ceriantharia (Anthozoa, Cnidaria). Hydrobiologia.

[ref-44] Nyholm KG (1943). Zur Entwicklung und Entwicklungsbiologie der Ceriantharien und Aktinien. Zoologiska bidrag från Uppsala.

[ref-45] Ocean Biodiversity Information System (OBIS) (2022). https://obis.org/taxon/100779.

[ref-46] Parker GH (1916). Locomotion of sea-anemones. Zoology.

[ref-47] Pennati R, Dell’Anna A, Pagliara P, Scarì G, Piraino S, De Bernardi F (2013). Neural system reorganization during metamorphosis in the planula larva of *Clava multicornis* (Hydrozoa, Cnidaria). Zoomorphology.

[ref-48] Perrier E (1893). Traité de zoologie: priméire partie—Zoologie génèrale protozoaires et phytózoaires arhtropodes.

[ref-49] Picton BE, Manuel RL (1985). Arachnanthus sarsi (Carlgren, 1912): a redescription of a cerianthid anemone new to the British Isles. Zoological Journal of the Linnean Society.

[ref-50] Piraino S, De Vito D, Schmich J, Bouillon J, Boero F (2004). Reverse development in Cnidaria. Canadian Journal of Zoology.

[ref-51] Piraino S, Zega G, Di Benedetto C, Leone A, Dell’Anna A, Pennati R, Carnevali DC, Schmid V, Reichert H (2011). Complex neural architecture in the diploblastic larva of *clava multicornis* (hydrozoa, cnidaria). The Journal of Comparative Neurology.

[ref-52] Rap WL (1829). Ueber die Polypen im Allgemeinen und die Actinien insbesondere. Naturhistorischer Versuch.

[ref-53] Roule L (1904). Sur un cèrianthaire nouveau. Comptes Rendus de l’Académie des Sciences.

[ref-54] Sars M, Sars M (1846). Ueber *Arachnactis albida*, einen schwimmenden Polypen. Fauna littoralis Norvegiae oder Beschreibung und Abbildungen neuer oder wenig bekannten, Seethiere, nebst Beobachtungen über die Organisation, Lebensweise und Entwickelung derselben.

[ref-55] Spano C, Flores V (2013). Staining protocol for the histological study of sea anemones (Anthozoa: Actiniaria) with recommendations for anesthesia and fixation of specimens. Latin American Journal of Aquatic Research.

[ref-56] Spier D, Stampar SN, Prantoni AL (2012). New record of the endangered cerianthid *Ceriantheomorphe brasiliensis* (Cnidaria: Hexacorallia) in Paranaguá Bay, southern Brazil. Marine Biodiversity Records.

[ref-57] Stampar SN, Beneti JS, Acuña FH, Morandini AC (2015a). Ultrastructure and tube formation in Ceriantharia (Cnidaria, Anthozoa). Zoologischer Anzeiger.

[ref-58] Stampar SN, El Didi SO, Paulay G, Berumen ML (2018). A new species of *Arachnanthus* from the Red Sea (Cnidaria, Ceriantharia). Zookeys.

[ref-59] Stampar SN, Maronna MM, Reimer JD, Beneti JS, Morandini AC, Goffredo S, Dubinsky Z (2016). Ceriantharia in current systematics: life cycles, morphology and genetics. The Cnidaria, past, present and future: the world of medusa and her sisters.

[ref-60] Stampar SN, Maronna MM, Vermeij MJA, Silveira FL, Morandini AC (2012). Evolutionary diversification of banded tube—dwelling anemones (Cnidaria; Ceriantharia; *Isarachnanthus*) in the Ocean Atlantic. PLOS ONE.

[ref-61] Stampar SN, Morandini AC, Branco LC, Silveira FL, Migotto AE (2015b). Drifting in the oceans: *Isarachnanthus nocturnus* (Cnidaria, Ceriantharia, Arachnactidae), an anthozoan with an extended planktonic stage. Marine Biology.

[ref-62] Stampar SN, Morandini AC, Silveira FL (2014). A new species of *Pachycerianthus* (Cnidaria, Anthozoa, Ceriantharia) from tropical southwestern Atlantic. Zootaxa.

[ref-63] Stampar SN, Reimer JD, Maronna MM, Lopes CSS, Ceriello H, Santos TB, Acuña FH, Morandini AC (2020). Ceriantharia (Cnidaria) of the world: an annotated catalogue and key to species. Zookeys.

[ref-64] Teixeira-Amaral P, Lemos PR, Muxagata E, Nagata R (2021). Temporal dynamics of mesoplanktonic cnidarians in a subtropical estuary: Environmental drivers and possible trophic effects. Estuarine Coastal and Shelf Science.

[ref-65] Thomson SA, Pyle RL, Ahyong ST, Alonso-Zarazaga M, Ammirati J, Araya JF, Ascher JS, Audisio TL, Azevedo-Santos VM, Bailly N, Baker WJ, Balke M, Barclay MVL, Barrett RL, Benine RC, Bickerstaff JRM, Bouchard P, Bour R, Bourgoin T, Boyko CB, Breure ASH, Brothers DJ, Byng JW, Campbell D, Ceríaco LMP, Cernák I, Cerretti P, Chang C-H, Cho S, Copus JM, Costello MJ, Cseh A, Csuzdi C, Culham A, D’Elía G, d’Udekem d’Acoz C, Daneliya ME, Dekker R, Dickinson EC, Dickinson TA, Dijkstra P, Paul van Dijk K-DB, Dima B, Dmitriev DA, Duistermaat L, Dumbacher JP, Eiserhardt WL, Ekrem T, Evenhuis NL, Faille A, Fernández-Triana JL, Fiesler E, Fishbein M, Fordham BG, Freitas AVL, Friol NR, Fritz U, Frøslev T, Funk VA, Gaimari SD, Garbino GST, Garraffoni ARS, Geml J, Gill AC, Gray A, Grazziotin FG, Greenslade P, Gutiérrez EE, Harvey MS, Hazevoet CJ, He K, He X, Helfer S, Helgen KM, Van Heteren AH, Garcia FH, Holstein N, Horváth MK, Hovenkamp PH, Hwang WS, Hyvönen J, Islam MB, Iverson JB, Ivie MA, Jaafar Z, Jackson MD, Pablo Jayat J, Johnson NF, Kaiser H, Klitgøard BB, Knapp DG, Kojima J.-I, Köljalg U, Kontschán J, Krell F-T, Krisai-Greilhuber I, Kullander S, Latella L, Lattke JE, Lencioni V, Lewis GP, Lhano MG, Lujan NK, Luksenburg JA, Mariaux J, Marinho-Filho J, Marshall CJ, Mate JF, McDonough MM, Michel E, Miranda VFO, Mitroiu M-D, Molinari J, Monks S, Moore AJ, Moratelli R, Murányi D, Nakano T, Nikolaeva S, Noyes J, Ohl M, Oleas NH, Orrell T, Páll-Gergely B, Pape T, Papp V, Parenti LR, Patterson D, Pavlinov IY, Pine RH, Poczai P, Prado J, Prathapan D, Rabeler RK, Randall JE, Rheindt FE, Rhodin AGJ, Rodríguez SM, Rogers DC, Roque FDO, Rowe KC, Ruedas LA, Salazar-Bravo J, Salvador RB, Sangster G, Sarmiento CE, Schigel DS, Schmidt S, Schueler FW, Segers H, Snow N, Souza-Dias PGB, Stals R, Stenroos S, Stone RD, Sturm CF, Štys P, Teta P, Thomas DC, Timm RM, Tindall BJ, Todd JA, Triebel D, Valdecasas AG, Vizzini A, Vorontsova MS, De Vos JM, Wagner P, Watling L, Weakley A, Welter-Schultes F, Whitmore D, Wilding N, Will K, Williams J, Wilson K, Winston JE, Wüster W, Yanega D, Yeates DK, Zaher H, Zhang G, Zhang Z-Q, Zhou HZ (2018). Taxonomy based on science is necessary for global conservation. PLOS Biology.

[ref-66] Tiffon Y (1987). Ordre des Cérianthaires. Traité de zoologie: anatomie, systematique, biologie - Cnidaires / Anthozoaires.

[ref-67] Uchida H (1979). Cerianthids (Anthozoa, Coelenterata) from Kii Region, Middle Japan. Bulletin of the National Science Museum.

[ref-68] Van Beneden E (1897). Die Anthozoen der Plankton-Expedition. Ergebnisse der Plankton-Expedition der Humboldt-Stiftung.

[ref-69] ØVan Beneden E (1924). Travaux posthumes d’Edouard Van Beneden sur les cérianthaires collationnés par Paul Cerfontaine. Archives of Biological Sciences.

[ref-70] Verrill AE (1865). Classification of polyps (extract condensed from a synopsis of the polypi of the North Pacific exploring expedition, under captains Ringgold and Rodgers, U.S.N.). Proceedings of the Essex Institute.

